# ICMT supports BRAF^V600E^-driven tumor growth by membrane targeting of the CAAX protein INPP5E

**DOI:** 10.1073/pnas.2601795123

**Published:** 2026-05-13

**Authors:** Xijie Yang, Xi Qiao, Sarah Schmidt, Ella A. Eklund, Carolina Ebner-Walter, Emil Ivarsson, Michelle Ann Crespo, Nadieh Kersemakers, Jozefina J. Dzanan, Bjarni Thorisson, Christine Lundgren, Hooi Ching Lim, Sam Shkoukani, Elin Tüksammel, Xiufeng Xu, Volkan I. Sayin, Mar Martín-Fontecha, Silvia Ortega-Gutiérrez, Martin Dalin, Martin O. Bergo

**Affiliations:** ^a^ Department of Medicine, Huddinge, Karolinska Institutet, Huddinge SE-141 83, Sweden; ^b^ Department of Surgery, Institute of Clinical Sciences, University of Gothenburg, Gothenburg SE-413 45, Sweden; ^c^ Sahlgrenska Center for Cancer Research, University of Gothenburg, Gothenburg SE-405 30, Sweden; ^d^ Department of Molecular and Clinical Medicine, Institute of Medicine, University of Gothenburg, Gothenburg SE-405 30, Sweden; ^e^ Organic Chemistry Department, Faculty of Optics and Optometry, Universidad Complutense de Madrid, Madrid 28040, Spain; ^f^ Organic Chemistry Department, Faculty of Chemical Sciences, Universidad Complutense de Madrid, Madrid 28040, Spain; ^g^ Department of Pediatrics, Institute of Clinical Sciences, University of Gothenburg, Gothenburg SE-416 85, Sweden

**Keywords:** CAAX protein processing, ICMT, BRAFV600E-driven cancer, INPP5E

## Abstract

Isoprenylcysteine carboxyl methyltransferase (ICMT) catalyzes C-terminal methylation of prenylated CAAX proteins, a final processing step promoting membrane association and signaling. Although ICMT has been pursued to disrupt RAS membrane targeting, its role in BRAF^V600E^-driven cancers and critical substrates remains unclear. Here, genetic and pharmacologic (UCM-1336) ICMT inhibition suppressed proliferation and invasion in BRAF^V600E^-mutant melanoma cells and reduced tumor growth in xenografts and mice. ICMT knockdown inhibited proliferation of BRAF-inhibitor-resistant melanoma cells. We identify INPP5E as an ICMT-dependent substrate: ICMT inhibition reduced INPP5E methylation, displaced it from membranes, and increased PI(4,5)P_2_. Forced INPP5E membrane targeting partially rescued growth defects caused by ICMT inhibition. These findings implicate an ICMT-INPP5E-axis that supports BRAF^V600E^-driven tumor growth.

Proteins with a C-A-A-X motif undergo three processing steps: cysteine prenylation, cleavage of the –AAX, and prenylcysteine methylation by isoprenylcysteine carboxyl methyltransferase (ICMT) (1, 2). This methylation enhances CAAX protein membrane association and fine-tunes signaling (e.g., rat sarcoma virus (RAS)). Because oncogenic RAS-signaling depends on subcellular localization, ICMT is considered an anticancer target, but its tumor-context dependencies and relevant substrates are incompletely defined (1).

*Icmt* inactivation can suppress Kirsten rat sarcoma virus oncogene (KRAS)-driven disease and early ICMT-inhibitors showed antiproliferative activity (3–5). However, *Icmt* loss can also accelerate progression in a KRAS-driven pancreatic cancer model (6), highlighting the need to define tumor contexts and substrates that mediate response. More selective inhibitors, including UCM-1336, show in vivo activity in RAS-driven acute myeloid leukemia and glioblastoma models, supporting pharmacologic ICMT inhibition (5, 7, 8).

B-Raf proto-oncogene, serine/threonine kinase (BRAF) mutations occur in ~8% of tumors and ~50% of melanomas (often BRAF^V600E^). Although MAPK/ERK kinase (MEK)-inhibitors can induce rapid clinical responses, resistance typically develops, motivating the search for complementary vulnerabilities.

We previously found that *Icmt* knockout blocks BRAF^V600E^-driven fibroblast transformation, despite BRAF itself lacking a CAAX motif, suggesting that one or more ICMT-dependent CAAX proteins enable BRAF^V600E^-driven tumorigenesis (9). Here we test genetic and pharmacologic ICMT inhibition in BRAF^V600E^-driven cancer models and human melanoma cells and identify inositol polyphosphate-5-phosphatase E (INPP5E) as an ICMT-dependent effector whose membrane targeting contributes to tumor growth.

## Results and Discussion

### Genetic and Pharmacologic ICMT Suppression Inhibits BRAF^V600E^-Driven Oncogenesis.

**Fig. 1.**
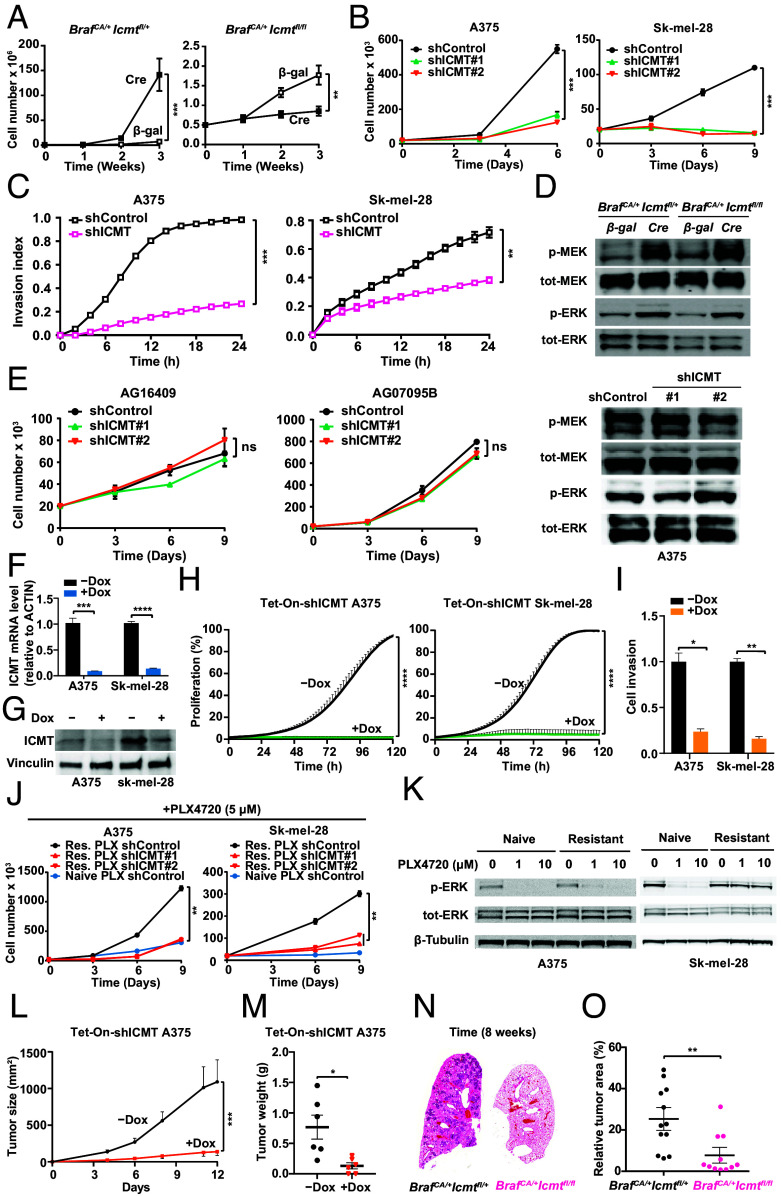
ICMT knockdown inhibits BRAF^V600E^-mutant melanoma growth in vitro and in vivo. (*A*) Proliferation of MEFs from *Braf^CA/+^Icmt^fl/+^* and *Braf^CA/+^Icmt^fl/fl^* embryos after infection with *Ad-βgal* or *Ad-Cre.* (*B*) Proliferation of BRAF^V600E^-mutant human melanoma cells after lentiviral transduction with shControl or shICMT. (*C*) Invasion of BRAF^V600E^-mutant human melanoma cells measured by xCELLigence real-time invasion assay. (*D*) Western blots of phospho-MEK, total MEK, phospho-ERK, and total ERK in *Ad-βgal*– or *Ad-Cre*–treated MEFs from panel A (*Upper*) and in A375 cells transduced with shControl or two independent shICMT constructs from panel B (*Lower*). (*E*) Proliferation of nontransformed human fibroblasts after lentiviral transduction with shControl or shICMT. (*F*) ICMT mRNA levels by qRT-PCR in A375 and Sk-mel-28 cells stably expressing Tet-On-shICMT 3 d after doxycycline (1 μg/mL). Data are mean values from triplicates. (*G*) Western blots of ICMT in A375 and Sk-mel-28 cells stably expressing Tet-On-shICMT 3 d after doxycycline (1 μg/mL). (*H*) Proliferation of Tet-On-shICMT A375 and Sk-mel-28 cells after doxycycline (1 μg/mL, 3 d), assessed with IncuCyte live-cell imaging. (*I*) Transwell invasion of Tet-On-shICMT A375 and Sk-mel-28 cells after doxycycline. (*J*) Proliferation of naïve and PLX4720-resistant BRAF^V600E^-mutant melanoma cells treated with PLX4720 (5 µM) after shRNA induction. (*K*) Western blot of phospho-ERK and total ERK in naïve and PLX4720-resistant melanoma cells treated with PLX4720. (*L* and *M*) Tumor growth (*L*) and end-point tumor weight (*M*) of Tet-On-shICMT A375 xenografts in NOD scid gamma (NSG) mice. Doxycycline (2 mg/mL in 5% sucrose) was provided in drinking water after tumors became palpable; tumor size was measured 2 to 3 times/wk (n = 6). (*N*) Representative H & E–stained lung sections from *Braf^CA/+^Icmt^fl/+^* and *Braf^CA/+^Icmt^fl/fl^* mice 8 wk after Ad-Cre inhalation. (*O*) Quantification of relative lung tumor area from panel *N* (n = 11 to 12).

We found that *Icmt* inactivation blocks transformation of BRAF^V600E^-overexpressing mouse embryonic fibroblasts (MEFs) (9). To test endogenous BRAF^V600E^, we isolated MEFs from *Braf^CA/+^Icmt^fl/+^* and *Braf^CA/+^Icmt^fl/fl^* embryos and activated BRAF^V600E^—and inactivated one or both *Icmt* alleles—using Cre-adenovirus (Ad-Cre). As expected, BRAF^V600E^ activation increased MEF proliferation, whereas *Icmt* inactivation abolished this proliferative advantage (Fig. 1*A*).

In BRAF^V600E^-mutant human melanoma cells, lentiviral ICMT knockdown reduced proliferation and invasion in xCELLigence assays (Fig. 1 *B* and *C*), without reductions in phospho-MEK or phospho-ERK (Fig. 1*D*). Notably, ICMT knockdown did not reduce proliferation of nontransformed human fibroblasts (Fig. 1*E*). Using doxycycline (dox)-inducible shRNAs, ICMT suppression reduced melanoma proliferation/invasion by IncuCyte and Matrigel assays (Fig. 1 *F*–*I*). ICMT knockdown also reduced proliferation of PLX4720-resistant BRAF^V600E^ melanoma derivatives with distinct resistance features (Fig. 1 *J* and *K*), indicating that acquired BRAF-inhibitor resistance does not eliminate ICMT vulnerability.

In subcutaneous xenografts carrying dox-inducible ICMT shRNAs, dox markedly reduced tumor growth (Fig. 1 *L* and *M*). To test endogenous tumor initiation, we administered Ad-*Cre* to activate BRAF^V600E^ in lung epithelium of *Braf^CA/+^Icmt^fl/+^* versus *Braf^CA/+^Icmt^fl/fl^* mice; *Icmt* deficiency significantly reduced lung tumor burden at 8 wk (Fig. 1 *N* and *O*). Together, these findings identify a dependence of BRAF^V600E^-driven tumors on ICMT across fibroblast, melanoma, and lung tumor contexts, without measurable mitogen-activated protein kinase (MAPK) suppression, consistent with a mechanism distinct from BRAF-MEK-ERK inhibition.

**Fig. 2.**
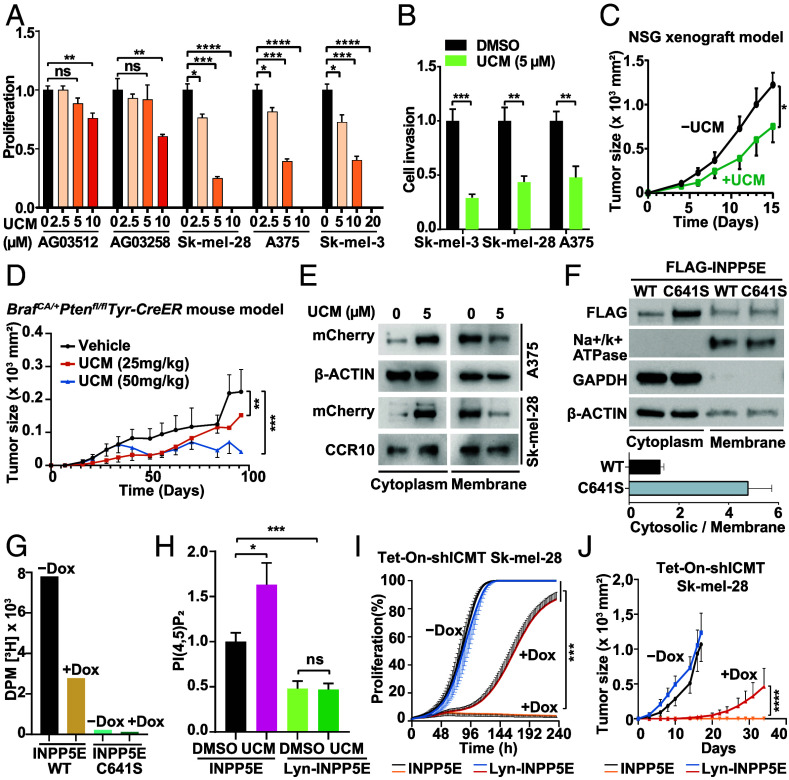
Pharmacologic ICMT inhibition with UCM-1336 suppresses BRAF^V600E^-mutant melanoma growth and implicates INPP5E. (*A*) Cell counts of fibroblasts (AG03512, AG03258) and melanoma cells (Sk-mel-28, A375, Sk-mel-3) after 3 d treatment with increasing concentrations of UCM-1336 (UCM) (n = 3). (*B*) Transwell invasion of Sk-mel-28, A375, Sk-mel-3 cells pretreated with UCM-1336 (5 μM, 24 h). (*C*) Tumor growth of A375 xenografts in NSG mice treated intraperitoneally with vehicle (n = 6) or UCM-1336 (25 mg/kg; n = 5) twice weekly for 2 wk; tumor size was measured 2 to 3 times/wk. (*D*) Tumor growth in tamoxifen-induced *Braf^CA/+^Pten^fl/fl^Tyr-CreER^+/0^*mice treated intraperitoneally with vehicle (n = 9), UCM-1336 (25 mg/kg; n = 12), or UCM-1336 (50 mg/kg; n = 11) twice weekly after tumors became palpable; tumor size was measured 2 to 3 times/wk. (*E*) Western blot of exogenous mCherry-INPP5E in A375 and Sk-mel-28 cells at baseline and after UCM-1336 (5 μM, 24 h). (*F*, *Upper*) Western blot of FLAG-INPP5E(WT) and FLAG-INPP5E(C641S) in cytosolic and membrane fractions. (*Lower*) Quantification of cytosolic/membrane distribution. (*G*) Metabolic labeling–based carboxyl-methylation assay in Tet-On-shICMT Sk-mel-28 cells expressing FLAG-INPP5E(WT) or FLAG-INPP5E(C641S) after doxycycline (1 μg/mL, 3 d). (*H*) Immunofluorescence intensity of plasma membrane PI(4,5)P_2_ in Sk-mel-28 cells expressing INPP5E or Lyn-INPP5E after UCM-1336 (5 μM, 24 h). (*I*) Proliferation of Tet-On-shICMT Sk-mel-28 cells expressing INPP5E or Lyn-INPP5E, with or without doxycycline (1 μg/mL), monitored by IncuCyte live-cell imaging (n = 2). (*J*) Tumor growth in NSG mice injected subcutaneously with Tet-On-shICMT Sk-mel-28 cells expressing INPP5E or Lyn-INPP5E. Doxycycline (2 mg/mL in 5% sucrose) was provided starting 3 d before injection; tumors were measured 2 to 3 times per week (n = 6 to 7).

We next used the selective ICMT inhibitor UCM-1336. In BRAF^V600E^-melanoma cells, UCM-1336 dose-dependently reduced proliferation/cell numbers and invasion, whereas nontransformed fibroblasts were minimally affected (Fig. 2 *A* and *B*). UCM-1336 also reduced growth of BRAF^V600E^-melanoma xenografts (Fig. 2*C*) and suppressed tumor growth in a *Cre*-inducible melanoma model driven by BRAF^V600E^ activation with *Pten* loss in melanocytes (Fig. 2*D*). The modest effects in fibroblasts under matched conditions suggest a therapeutic window, addressing concern that CAAX processing enzymes might be broadly required in normal cells.

### ICMT-Dependent Membrane Targeting of INPP5E Supports Melanoma Growth.

Because ICMT suppression did not reduce MAPK signaling [Fig. 1*D*, (9)], we asked whether other CAAX proteins mediate the antitumor effects. Exploratory transcriptomic profiling revealed cilia-associated genes, prompting examination of INPP5E, an inositol polyphosphate 5-phosphatase.

Consistent with ICMT-dependent membrane targeting, UCM-1336 shifted mCherry-INPP5E from membranes toward the cytosol (Fig. 2*E*). A prenylation-defective INPP5E mutant (C641S) similarly accumulated in the cytosolic fraction (Fig. 2*F*). In Tet-on-shICMT-Sk-mel-28 cells, metabolic labeling showed reduced base-labile methyl incorporation into FLAG-INPP5E(WT) upon dox-induced ICMT knockdown (Fig. 2*G*). Functionally, UCM-1336 increased cellular PI(4,5)P_2_ in cells expressing INPP5E (WT), but not in cells expressing forced membrane-targeted INPP5E (Lyn-INPP5E) (Fig. 2*H*). This plasma-membrane PI(4,5)P_2_ increase is consistent with reduced membrane-associated INPP5E activity and may contribute to the growth and invasion defects by altering phosphoinositide-dependent signaling, although downstream effectors remain undefined. Finally, Lyn-INPP5E partially restored proliferation during ICMT suppression in vitro and xenograft growth in vivo (Fig. 2 *I* and *J*), which places INPP5E downstream of ICMT and implies contributions from additional ICMT substrates.

Two translational points are notable. First, nontransformed fibroblasts were less sensitive than melanoma cells to ICMT inhibition, suggesting a therapeutic window. Second, the strongest clinical opportunity is likely combination therapy rather than monotherapy. Although ICMT is an immature target and on-target liabilities may emerge with greater potency (1, 10), ICMT suppression inhibits BRAF-inhibitor-resistant models without MAPK suppression, indicating a distinct, potentially complementary mechanism. Testable combinations include ICMT inhibition with BRAF/MEK-inhibitors to delay/overcome resistance; with PI3K/AKT targeting in PTEN-deficient disease; or with immunotherapy if ICMT/INPP5E-dependent phosphoinositide remodeling influences tumor–immune interactions (9, 11, 12). These findings suggest that ICMT-dependent CAAX processing is a vulnerability in BRAF^V600E^-driven cancer and identify INPP5E membrane targeting as an effector.

### Method Summary.

We tested genetic and pharmacologic ICMT inhibition (UCM-1336) in BRAF^V600E^-driven mouse models and human melanoma cells. We assessed oncogenic phenotypes. Key assays were protein methylation, membrane partitioning, and phosphoinositide measurements. Details in *SI Appendix*.

## Supplementary Material

Appendix 01 (PDF)

## Data Availability

All study data are included in the article and/or *SI Appendix*.
