# Supplementary material for: ICMT supports BRAFV600E-driven tumor growth by membrane targeting of the CAAX protein INPP5E
Source: Proc Natl Acad Sci U S A. 2026 May 13;123(20):e2601795123. doi: 10.1073/pnas.2601795123 (PMC13187791; doi:10.1073/pnas.2601795123)
Supplement: Supplementary file 1 — Appendix 01 (PDF) [file pnas.2601795123.sapp.pdf]

Supporting Information

SI Methods

Mice. Conditional $\textit{Braf}^{CA}$ *and* $\textit{Icmt}^{fl}$ alleles were described previously (1, 2). Lung tumors were initiated in $\textit{Braf}^{CA/+}\textit{Icmt}^{fl/+}$ and $\textit{Braf}^{CA/+}\textit{Icmt}^{fl/fl}$ mice by inhalation of *Cre*-adenovirus (Ad-*Cre*) at 8 weeks of age using established protocols (3); tumor burden was quantified on H&E–stained sections by area measurement. For endogenous melanoma, $\textit{Braf}^{CA/+}\textit{Pten}^{fl/fl}\textit{Tyr-CreER}^{+/0}$ (BPT) mice were administered tamoxifen at postnatal days 2–4 (4). Xenograft experiments were performed by subcutaneous injection of human melanoma cells into NSG (NOD.Cg-$\textit{Prkdc}^{\text{scid}}$ $\textit{Il2rg}^{\text{tm1Wjl}}$/SzJ) mice.

Cells and perturbations. Human melanoma lines and nontransformed fibroblasts were cultured in DMEM with 10% FBS; all lines were mycoplasma negative. ICMT was suppressed with lentiviral shRNAs or doxycycline-inducible shICMT. INPP5E(WT) and Lyn-INPP5E constructs were expressed from lentiviral vectors. PLX4720 resistance was generated by stepwise dose escalation. UCM-1336, used at the indicated concentrations, was synthesized as described (5) with satisfactory spectroscopic data and purity (>95% HPLC).

Assays. Proliferation was measured by cell counting and real-time imaging (IncuCyte) or impedance (xCELLigence). Invasion was quantified by Matrigel trans-well or xCELLigence CIM-plate assays. Viability after 24 h UCM-1336 exposure was measured by PrestoBlue. Protein levels and signaling were assessed by western blot and membrane/cytosol fractionation. INPP5E carboxyl methylation was measured by metabolic labeling with [^{3}H-methyl]-methionine followed by FLAG immunoprecipitation and base-labile [^{3}H]-methanol release (6). Plasma membrane PI(4,5)P_2 was quantified by immunofluorescence normalized to the plasma membrane marker Alexa Fluor 350-conjugated wheat germ agglutinin (WGA) (7).

In vivo drug studies. NSG xenograft-bearing mice (from 1×10^6 cells) received intraperitoneal UCM-1336 or vehicle twice weekly; tumor volume was calculated as ($\text{width}^2 \times \text{length}$)/2. BPT mice with palpable tumors likewise received intraperitoneal UCM-1336 or vehicle twice weekly at the indicated doses.

Statistics and ethics. We used two-tailed *t* tests or two-way ANOVA as appropriate; n and exact tests are stated in figure legends. Animal studies were ethically approved (Sweden).

SI References

1. M. O. Bergo, et al., Inactivation of Icmt inhibits transformation by oncogenic K-Ras and B-Raf. J. Clin. Invest. 113, 539–550 (2004).

2. D. Dankort, et al., A new mouse model to explore the initiation, progression, and therapy of BRAFV600E-induced lung tumors. Genes Dev. 21, 379–384 (2007).

3. M. Liu, et al., Targeting the protein prenyltransferases efficiently reduces tumor development in mice with K-RAS-induced lung cancer. Proceedings of the National Academy of Sciences 107, 6471–6476 (2010).

4. D. Dankort, et al., BrafV600E cooperates with Pten loss to induce metastatic melanoma. Nat. Genet. 41, 544–552 (2009).

5. N. I. Marín-Ramos, et al., A Potent Isoprenylcysteine Carboxylmethyltransferase (ICMT) Inhibitor Improves Survival in Ras-Driven Acute Myeloid Leukemia. J. Med. Chem. 62, 6035–6046 (2019).

6. H. Court, I. M. Ahearn, M. Amoyel, E. A. Bach, M. R. Philips, Regulation of NOTCH signaling by RAB7 and RAB8 requires carboxyl methylation by ICMT. Journal of Cell Biology 216, 4165–4182 (2017).

7. K. Kanemaru, et al., Plasma membrane phosphatidylinositol (4,5)-bisphosphate is critical for determination of epithelial characteristics. Nat. Commun. 13, 2347 (2022).
